# Developing an innovative medical ethics and law curriculum—constructing a situation-based, interdisciplinary, court-based learning course: a mixed methods study

**DOI:** 10.1186/s12909-022-03349-z

**Published:** 2022-04-15

**Authors:** Wan-Ting Chen, Chung-Pei Fu, Yan-Di Chang, Yi-Chih Shiao, Po-Yi Chen, Chih-Chia Wang

**Affiliations:** 1 Present Address: Department of Psychiatry, Tri-Service General Hospital, National Defense Medical Center, No.325, Sec. 2, Chenggong Rd., Neihu Dist, 11490 Taipei City, Taiwan; 2 School of Medicine, National Defense Medical Center, No.161, Sec. 6, Minquan E. Rd., Neihu Dist, 11490 Taipei City, Taiwan; 3 Department of Occupational Therapy, College of Medicine, Fu Jen Catholic University, No.510, Zhongzheng Rd., Xinzhuang Dist, New Taipei City, Taiwan; 4 Center of Medical Humanities Education, School of Medicine, National Defense Medical Center, No.161, Sec. 6, Minquan E. Rd., Neihu Dist, 11490 Taipei City, Taiwan; 5 Present Address: Department of Family and Community Medicine, Tri-Service General Hospital, National Defense Medical Center, No.325, Sec. 2, Chenggong Rd., Neihu Dist, 11490 Taipei City, Taiwan; 6 College of Law, National Chengchi University, No. 64, Sec. 2, Jhihnan Rd., Wunshan Dist., Taipei City, 11605 Taiwan

**Keywords:** Interdisciplinary, Court and litigation, Medical law education, Situation-based learning

## Abstract

**Background:**

Traditional lecture-based medical ethics and law courses deliver knowledge but may not improve students’ learning motivation. To bridge this theory-to-practice gap and facilitate students’ learning effectiveness, we applied situated-learning theory to design an interdisciplinary court-based learning (CBL) component within the curriculum. Our study aimed to investigate students’ learning feedbacks and propose a creative course design.

**Methods:**

A total of 135 fourth-year medical students participated in this course. The CBL component included 1 h of introduction, 1 h of court attendance, and 2 h of interdisciplinary discussion with senior physicians, judges, and prosecutors. After the class, we conducted a survey using a mixed-methods approach to gauge students’ perceptions of engagement, performance, and satisfaction.

**Results:**

A total of 97 questionnaires were received (72% response rate). Over 70% of respondents were satisfied and felt that the class was useful except for role-playing activities (60%). More than 60% reported a better understanding of the practical applications of medical law. Approximately half (54%) reported less anxiety about medical disputes. 73% reported that the lecture provided awareness of potential medical disputes, and most respondents expressed an interest in medical law courses after the court visit (78%). 80% of the respondents were able to display empathy and apply mediation skills. Qualitative analyses showed that students demonstrated new knowledge, including recognizing the significance of the medical profession, distinguishing the importance of physician-patient communication, having confidence in the fairness of the justice system, and being willing to increase their legal knowledge.

**Conclusions:**

CBL curriculum increases students’ learning motivation in strengthening medical professionalism and medical law, develops students’ empathy for patients and communication skills, as well as builds up students’ trust in the justice system. This novel course design can be applied to teach medical ethics and law.

**Supplementary information:**

The online version contains supplementary material available at 10.1186/s12909-022-03349-z.

## Background

Developing interdisciplinarity in medical ethics and law is an important issue in the current medical education system [1–3], especially under the recent circumstances where there has been a surge in medical disputes and tense doctor-patient relationships [4]. In fact, investigations have shown an inverse relationship between the lack of medical law knowledge and legal defensiveness [5, 6]. Nonetheless, legal knowledge gaps continue to exist among health professions, indicating a paucity in effective medical law education [7, 8]. One study showed that physicians felt uncomfortable with deficient knowledge on medical malpractice [9]. Indeed, the need for programmatic development in medical law education should be highlighted [10], with multidisciplinary ethical education being one approach toward bridging the gap [8].

The appropriate customization of teaching materials is crucial for medical education from the perspective of international medico-legal education considering that attitudes toward medical disputes vary among countries [5]. For instance, physicians in the East tend to be more aggrieved when encountering in medical disputes, which may be due to an innate attitude of conflict avoidance. Unlike Westerners who prefer to communicate forthrightly, Asians tend to endure dissatisfaction to attain temporary peace [11]. Moreover, settlements in litigation differ between each country. In Taiwan, patients who fail to achieve reconciliation with the hospital following a medical dispute will be compulsorily referred to mediation before entering judicial proceedings (Figure S1 in the Supplementary Appendix). Thus, to design a course that suits the needs of the local culture and society, multifaceted strategies based on sound pedagogies should be adopted [12, 13].

The mainstream method for teaching medical ethics and law involves direct instruction through lectures [14], which may easily fail to motivate students to learn medical ethics and law [13]. Moreover, conventional medical ethics and law courses are often taught by a combination of physicians and general education teachers instead of healthcare law specialists, which subsequently generates an incoherent curriculum. Therefore, to integrate the theoretical and practical aspects of medical ethics and law and enhance students’ ability to confront ethical dilemmas, an increasing number of schools have developed multidisciplinary medico-legal education [1]. For instance, the medical-legal partnership in Atlanta trains future physicians to coordinate with lawyers to help improve the health status and legal rights of their patients [15]. This reciprocal interaction between doctors and lawyers improves interprofessional collaboration [16, 17].

To develop a novel interdisciplinary medical law course, we applied situated-learning theory (SLT) to create the first court-based learning (CBL) component. Lave and Wenger’s SLT helps students construct knowledge and promote learning reflection by providing real cases and experiences wherein students interactively learn from experts [18]. SLT has been applied in healthcare-related fields, such as in core medical courses [19], clinical practice courses [20, 21], faculty development programs [22], and medical ethics and law courses [23]. Through this CBL component, students were able to visit a real court and interactively learn from actual cases with experts. The primary goal of our study was to use a mixed-methods evaluation approach to examine students’ affective and cognitive perceptions of their learning experience.

## Methods

### Participants

 Participants of the study were fourth-year medical students at the National Defense Medical Center in Taiwan who had completed their preclinical courses and were awaiting to begin their clerkship. They attended a compulsory 1-credit (18 h) Medical Ethics and Law course to obtain fundamental knowledge of medical legislation and mediation of medical disputes, with the final portion of the course consisting of a 4-hour CBL.

### Instruments

A semi-structured questionnaire (Table S1) was designed for evaluation after CBL. It composed of both open- and closed-ended questions, including 18 questions for students to rate their (1) satisfaction, (2) view of its practicality, (3) gain in knowledge, attitudes, and skills toward CBL, and open-ended comments about their experience and opinion about what they had learned and what impressed them most. The instructor in-charge gave explicit instructions for anonymity including setting up a deadline of 3 days for receiving feedback, ways to ensure students’ anonymity, reassuring them that their feedback was not tied to their grades, and providing tips for completing the two-part questionnaire. Our teaching assistant helped with data collection, identification removal, and screening of eligibility of feedback. Feedback with no content, contents beyond the confines of medicine/ethics/law, and those who did not meet the 3-day deadline was regarded as invalid. Contents of the remaining eligible questionnaires were then analyzed.

### Procedures

CBL encompassed 1 h of introduction, 1 h of court attendance, and 2 h of panel discussion (Table 1). The introduction provided students with basic knowledge of the processes of the court and the roles of the different parties. For the latter, students experienced what it was like for various parties through role-playing, for example, witnesses providing testimony, the prosecutor and lawyers debating their viewpoints, and the judge dispensing the sentence. Next, students were assigned to attend various proceedings in the public court, such as civil and criminal cases, state compensation, cases concerning insider trading, etc. During this process, students learned about the different roles in the litigation, the procedures, and skills in questioning and answering. Finally, they participated in an interdisciplinary panel discussion, in which senior judges, prosecutors, and physicians from the Taiwan Medical Association and Tri-Service General Hospital (TSGH) answered their queries and shared interesting insights and experiences in mediation with the students. Mediation was highlighted as an important alternative to litigation after the occurrence of a medical dispute that involved much communication and negotiation. The judges and prosecutors shared how they dealt with medical lawsuits and the status of medical litigation, whereas physicians explained what students should pay attention to in their future practice to avoid medical disputes. A senior judge even told the students about a revolutionary mechanism for dispute resolution called alternative dispute resolution. After the class, students completed a voluntary and anonymous questionnaire (Table S1). The institutional review board of TSGH approved this study (TSGHIRB: 1-106-05-027).

**Table 1:** Parts of the court-based learning component in the medical ethics and law course

Part 1: Introduction (1 h)
• Consists of two parts:
I. Introduction to the court, Taiwan’s judicial system and its levels of trials, the rights and obligations of all parties, and court proceedings
II. Role-play: students assume various roles in a lawsuit, from the judge, prosecutor, lawyer, plaintiff, defendant, to witness, and put on the proper court dresses accordingly
Part 2: Audit a court case (1 h)
• Students attend cases on trial that day
Part 3: Panel discussion
• With 2–3 senior judges, 2–3 senior prosecutors, and 2–3 senior physicians
• Consists of two parts:
I. Question and answer: students ask any questions, including those unrelated to the court visit
II. Sharing of experience: experts share their wisdom about the mediation process

### Data analyses

We applied mixed-methods evaluation approach to analyze our data [24]. Quantitative data were analyzed using SPSS software, whereas qualitative information was analyzed using thematic analysis [25] and a general inductive approach described below [26, 27]: the five longest responses were selected and read by two researchers with training in medical professionalism and medical law to find meaningful words and phrases related to the purpose of this research for preliminary coding. Next, the researchers compared, discussed, and integrated each other’s codes to form an initial classification. If there was a disagreement, a third researcher reviewed it. The process was repeated until consensus was reached, and no new codes emerged. A classification code was compiled for all three researchers to analyze the remaining data individually. At various checkpoints, the researchers reviewed and discussed the results. Qualitative data was translated from Chinese to English and proofread by native English speakers to ensure the precision of the original text.

## Results

### Results of quantitative analyses

Our screening process is summarized into a flow chart in the supplementary appendix (Figure S2). Out of the 135 students, data from 38 students (28%) were deemed invalid, including missing feedback in 23 students (17%), missing response content in 2 students (2%), missing the deadline in 6 students (4%), and contents beyond the confines of medicine/ethics/law in 7 students (5%). The response rate was 72%.

Over 70% of respondents were satisfied or highly satisfied with the CBL experience. For each component, 89% were satisfied or highly satisfied with the basic introduction, 89% with auditing a court case, 81% with role-playing, 79% with the ‘question and answer’ part in the panel discussion, and 77% with the ‘sharing of experience’ in the second part of the panel discussion. Over three-quarters of the students felt that CBL was useful or very useful, except for the role-playing activity (60%) (Table 2).

**Table 2:** Students’ self-evaluation of their satisfaction and views about the practicality of each part of their court-based learning

Satisfaction						
	Highly Dissatisfied (1) (n, %)	Dissatisfied (2) (n, %)	Neutral (3) (n, %)	Satisfied (3) (n, %)	Highly Satisfied (5) (n, %)	Mean
Introduction I	0 (0)	0 (0)	10 (11)	44 (47)	40 (42)	4.32 ± 0.66
Introduction II (role-play)	1 (1)	1 (1)	16 (17)	38 (40)	39 (41)	4.19 ± 0.83
Audit a court case	0 (0)	1 (1)	9 (10)	45 (51)	34 (38)	4.26 ± 0.68
Panel discussion I (Q&A)	0 (0)	3 (3)	16 (18)	42 (46)	30 (33)	4.09 ± 0.80
Panel discussion II (sharing of experience)	0 (0)	3 (3)	18 (20)	39 (43)	31 (34)	4.08 ± 0.82
Practicality						
	Not at All Useful (1) (n, %)	Not Useful (2) (n, %)	Neutral (3) (n, %)	Useful (4) (n, %)	Very Useful (5) (n, %)	Mean
Introduction I	0 (0)	1 (1)	12 (14)	45 (51)	31 (34)	4.19±0.71
Introduction II (role-play)	6 (7)	1 (1)	29 (32)	33 (37)	21 (23)	3.69±1.06
Audit a court case	1 (1)	0 (0)	19 (23)	33 (39)	31 (37)	4.11±0.84
Panel discussion I (Q&A)	2 (2)	1 (1)	16 (19)	42 (49)	25 (29)	4.01±0.86
Panel discussion II (sharing of experience)	1 (1)	0 (0)	18 (21)	42 (49)	25 (29)	4.05±0.78

More than 60% of the students reported a comprehensive understanding in knowledge, attitudes, and skills in medical law from the CBL experience. The item with the lowest percentage of agreement was “comprehended mediation” (67%) and that with the highest percentage of agreement was “understood the court operations” (74%). About half of the students had less anxiety about medical disputes (54%) and approximately one-third (31%) were neutral. This lecture increased students’ awareness of potential medical disputes (73%) and interest in medical law courses after the court visit (78%). Four-fifths (80%) of the students stated that they were better able to exhibit empathy and apply mediation skills in the future (Table 3).

**Table 3:** Students’ self-evaluation of their knowledge, attitudes, and skills after their court-based learning

	Do Not Understand at All (1) (n, %)	Do Not Understand (2) (n, %)	Neutral (3) (n, %)	Understand (4) (n, %)	Completely Understand (5) (n, %)	Mean
Court operations	0 (0)	1 (1)	24 (25)	62 (64)	10 (10)	3.84±0.61
Medical lawsuit	0 (0)	2 (2)	27 (28)	58 (60)	10 (10)	3.78±0.65
Mediation	0 (0)	4 (4)	28 (29)	52 (54)	12 (13)	3.75±0.73
	Strongly Disagree (1) (n, %)	Disagree (2) (n, %)	Neutral (3) (n, %)	Agree (4) (n, %)	Strongly Agree (5) (n, %)	Mean
Less worried about medical disputes	1 (1)	13 (14)	29 (31)	43 (46)	8 (8)	3.47±0.88
Accept the court’s ruling	1 (1)	6 (6)	28 (29)	51 (53)	10 (11)	3.66±0.79
Aware of potential medical disputes	0 (0)	2 (2)	24 (25)	56 (58)	14 (15)	3.85±0.68
Able to show empathy and apply mediation skills	0 (0)	0 (0)	19 (20)	61 (63)	16 (17)	3.97±0.61
More interested in medical law courses	0 (0)	1 (1)	20 (21)	54 (56)	21 (22)	3.99±0.69

### Results of qualitative analyses

In our analysis, most of the students wrote open-ended responses in a chronological sequence, especially when it came to their experiences throughout their lives. More than half of the participants mentioned how they came to know about the law in the past (e.g., from their parents with related jobs, self-interest, or similar visiting experiences) and what made them change during the process of immersing themselves into exploring knowledge of medical law through the instruction of those educators in-charge. Most of them had good prospects and aspirations about the mutual understanding between medicine and law for future work, whereas a few tended to be pessimistic about collaboration between the fields of medical and law fields. Through familiarity with the judicial system and the roles of various participants gained from firsthand observations, students realized the value of interdisciplinary collaboration between the medical and legal fields for the benefit of both doctors and patients.

In accordance with students’ responses collected, four distinct themes emerged:

1. Encourage students to sharpen their professional skills and pay attention to medical ethics and law.

Some of the respondents said that they realized that the medical profession was key to reducing lawsuits in clinical encounters. They understood the importance of upholding medical professionalism and following treatment guidelines to protect the rights of patients:

"As long as I treat my patients to the best of my professional knowledge and try my best to provide reasonable explanations (to my medical decisions), I can get good results (in avoiding medical disputes)".

Students also discovered that they had to always keep medical ethics and law in mind during practice and that being aware of medical jurisprudence could help reduce the occurrences of medical disputes as doctors. On the contrary, unfamiliarity with medical law would be a risk factor for potential medical disputes:

"As medical personnel, I need to pay more attention to relevant laws and regulations and abide by the rules to avoid lawsuits in the future".

2. Understand the importance of doctor-patient communication.

CBL provided students with a valuable opportunity for direct observation of medical litigation. Of the respondents, around one-quarter agreed that clear communication between doctors and patients was key to solid doctor-patient relationships:

"Through sharing and analyzing cases involving medical disputes, I understood the concerns of patients and what matters to them. After all, the patients’ perspectives may be very different from those of the doctors".

Furthermore, students mentioned that to prevent conflicts and reduce the gap in expectations between physicians and patients, physicians should show empathy and understanding toward their patients’ concerns:

"After explanations by medical and legal professionals in the discussion, I know how to seek assistance when a medical dispute occurs. I should also be careful with my words and avoid negative language when I talk to patients".

3. Establish trust in the judicial system.

The lack of proper exposure to medical jurisprudence and sensational reporting of medical disputes in the media led to more than one-quarter of the respondents reporting certain stereotypical images of the law and distrust in the ability of the justice system to determine fair judgment:

"Although we, as medical students, have not yet begun clinical work, we have already been exposed to the stress of medical disputes through exaggerated reports from the mass media. Medical disputes become ‘ghosts’ that persistently haunt us of the sinister work environment".

Observing litigation in an actual court allowed students to view the procedure and legal actions without sustaining any stress or pressure. Interactions with the legal personnel highlighted the similarities and differences between medicine and law:

"Through discussions with both medical and law experts, I identified dramatic differences in viewpoints between the medical staff and those legal advisers. It was beneficial but challenging",

By observing how the plaintiff and defendant debated their positions and addressed questions from the judge, students comprehended that every judicial decision was based on concrete evidence, ideally without bias or prejudice. They also witnessed that judges were not hostile to doctors. In fact, judges expended much effort to improve the litigation environment, including organizing medical law seminars, mediating medical disputes, and promoting relative legislation to optimize medical dispute processes:

"Though we may believe that our thinking is logical, we should not complain about the unfairness (of medical disputes) behind the scenes, but rather understand the viewpoint of the legal profession and speak out so they can understand us".

4. Improve students’ motivation to learn medical ethics and law.

More than one-quarter of respondents pointed out that the panel discussion provided a platform for different professionals to communicate and understand one another and supplied them with multidimensional thinking. Students mentioned that they had fewer negative emotions about medical disputes and a better understanding of medical law:

"I feel more relaxed and not that anxious about my (future) medical career and current medical working environment after I know about the types of medical disputes, what I should look out for, and how to prepare for and deal with them".

Students recognized that if the legal and medical professions did not cooperate, it would be impossible to mediate medical disputes. On the other hand, if these two professions worked together, the litigation environment would be improved greatly. After CBL, participants agreed that the medical profession should engage actively with the legal profession and exchange expert insights:

“I learned about what medical disputes are and how law professionals look upon doctors’ defense in lawsuits through face-to-face discussions with judges and prosecutors who deal with them,” and

"Attending the court session provided us (with the knowledge) so that we can be calm when we face litigations in the future since we have already seen what it is like. (This experience) may greatly reduce our tension if we have to go to court (later on)".

Moreover, students expressed their willingness to learn about medical jurisprudence, which would be beneficial in establishing an effective and amicable dispute-resolution process with cooperation between the fields of medicine and law:

"Initially, I thought that professional law terminologies were too difficult to understand, and I was a little bit repulsed and reluctant to learn,” “However, after visiting the court, I found that it was not boring at all",

## Discussion

Our CBL, which is an application of the social cognitive theory [28, 29], was designed to enhance preclinical medical students’ ability for multidisciplinary learning of medicine and law. Students gained more knowledge regarding the application of the law in a non-medical environment. As students observed judicial processes and interacted with legal personnel, they learned that professionalism is the cornerstone of medical practice. Beyond professionalism, they acknowledged that medical ethics and law help doctors become aware of their legal responsibilities, which not only protects them by reducing occurrences of medical disputes but also forms a crucial foundation for quality medical care. Students also agreed that good physician–patient communication played a key role in ameliorating the risk of lawsuits. Moreover, students eliminated negative stereotypes regarding the judicial system and increased their trust in its fair judgments through their CBL experience.

CBL is an innovative pedagogical method used in undergraduate medical law education. Unlike previous studies, which have mainly focused on medico-legal education and assessment among physicians-in-training (e.g. residents, fellows) [6, 30], we applied CBL to undergraduate education with the aim of empowering students to apply their theoretical knowledge in medical ethics and law to clinical practice and eliminating their preclinical anxiety about potential lawsuits. In addition, CBL demonstrates the value of cross-disciplinary communication [31]. Students were able to improve their legal thinking process without the need for complex legal knowledge through a didactic and interactive educational seminar. Direct contact with judicial personnel inspired students to focus on commonalities between medical and law professions rather than highlighting their differences [32], which strengthen the quality of teaching and ensure that students did not misunderstand what was being taught [33].

Our study indicated that students could self-reflect on their abilities as future doctors in non-medical courses outside the medical field. As students interacted with people with non-medical backgrounds, they understood that medical law could empower doctors to be proactive in securing their patients’ best interests, preventing malpractice and/or negligence, and avoiding civil or criminal consequences [34]. Students were able to acknowledge the importance of physician-patient communication in CBL despite having no direct dialogues with patients yet. As students observed court proceedings and discovered that defendants could barely understand what the judge and plaintiff said most of the time, they realized that inequity in understanding medical information also occurs in patients in medical encounters. This unique experience helped students realized that doctors should make more effort to address patients’ problems and anxieties instead of viewing patients as potential threats in medical litigations [35].

One of the significant findings that have emerged from the current study was that students reported having confidence in the judicial system after their CBL experience. A possible reason might be that the traditional medical education system did not address or ameliorate students’ skepticism and distrust of the justice system from unfavorable and biased media reports on medical litigations [36, 37], which indirectly deteriorates the relationship between doctors and patients, and drives more doctors to practice defensive medicine [35, 38]. CBL provides a platform that encourages students to voice their perspectives and break stereotypes. Moreover, students became motivated to learn medical ethics and law, highlighting the urgent need for more comprehensive instruction to students in medical jurisprudence.

Establishing the concept of medical law in medical education is indispensable [39, 40]; however, conventional teaching approaches do not allow students to sufficiently apply the law to medicine [41, 42]. Although medical ethics and law are equally emphasized in the curriculum statements of many medical schools, the actual teaching tends to focus on the former owing either to lack of faculty expertise or the inability to translate theory into practice. CBL is one of our pilot programs that aim at integrating local judicial systems into medical education to address the aforementioned problems. We invited judges to serve as teachers for CBL considering that most legal processes in Taiwan are based on litigation. The results of this research support the notion that medico-legal education should be tailored to the local legal culture. For countries that rely on a non-litigation system, arbitrators can be recruited as instructors to guide students in their learning. Other alternative strategies, including mock court practice and recruitment of both medical and law students in interprofessional courses [43], can be utilized to reinforce clinical relevance.

The study has some limitations: although our results indicated that students developed a positive attitude toward medico-legal problems, little could be inferred regarding their behavioral changes. Thus, longitudinal research is needed to determine students’ long-term development. Another limitation is the lack of an accurate assessment of the students’ knowledge and skills gained in medical law from the course. More research is required to objectively evaluate changes in students’ knowledge and attitudes.

## Conclusions

Strategies for the prevention of medical disputes, such as improving professionalism and patient communication, should be emphasized in medical law courses. CBL inspires students to learn and broaden their medical knowledge and improve their skills and attitudes in these areas. Moreover, this type of learning consolidates experiences in medical ethics and law, removing negative stereotypes about medical disputes and the justice system. This may result in the establishment of trust in the fairness of the justice system.

## Supplementary Information

**Additional file 1.**

## Data Availability

The datasets generated during and/or analyzed during the current study are available from the corresponding author on reasonable request.

## Abbreviations

SLT: Situated-learning theory
CBL: Court-based learning
TSGH: Tri-Service General Hospital
