# Supplementary material for: Developing an innovative medical ethics and law curriculum—constructing a situation-based, interdisciplinary, court-based learning course: a mixed methods study
Source: BMC Med Educ. 2022 Apr 15;22:284. doi: 10.1186/s12909-022-03349-z (PMC9011998; doi:10.1186/s12909-022-03349-z)
Supplement: Supplementary file 1 — Additional file 1. [file 12909_2022_3349_MOESM1_ESM.docx]

Supplementary Appendix

This appendix has been provided by the authors to give readers additional information about the work.

Supplement to: Wan-Ting Chen, Chung-Pei Fu, Yan-Di Chang, Yi-Chih Shiao, Po-Yi Chen, and Chih-Chia Wang. Developing an Innovative Medical Ethics and Law Curriculum—Constructing a Situation-Based, Interdisciplinary Court-Based Learning Course: A Mixed Methods Study

organization. BMC Med. Educ.

**Supplementary materials**

Developing an Innovative Medical Ethics and Law Curriculum—Constructing a Situation-Based, Interdisciplinary Court-Based Learning Course: A Mixed Methods Study

Wan-Ting Chen^a^, Chung-Pei Fu^b^, Yan-Di Chang^c^, Yi-Chih Shiao^d^, Po-Yi Chen^e^, and Chih-Chia Wang^f^*

*^a^Department of Psychiatry, Tri-Service General Hospital and School of Medicine, National Defense Medical Center, No.325, Sec. 2, Chenggong Rd., Neihu Dist., Taipei City, 11490, Taiwan, friend1584@gmail.com; ^b^Department of Occupational Therapy, College of Medicine, Fu Jen Catholic University, No.510, Zhongzheng Rd., Xinzhuang Dist., New Taipei City, Taiwan,* *cpfu0617@gmail.com; ^c^Center of Medical Humanities Education, School of Medicine, National Defense Medical Center, No.161, Sec. 6, Minquan E. Rd., Neihu Dist., Taipei City, 11490, Taiwan, changyandi@gmail.com; ^d^Department of Family and Community Medicine, Tri-Service General Hospital and School of Medicine, National Defense Medical Center, No.325, Sec. 2, Chenggong Rd., Neihu Dist., Taipei City, 11490, Taiwan, darthravenhugo@gmail.com; ^e^School of Medicine, National Defense Medical Center, No.161, Sec. 6, Minquan E. Rd., Neihu Dist., Taipei City, 11490, Taiwan, 96504034@nccu.edu.tw; ^f^Department of Family and Community Medicine, Tri-Service General Hospital and School of Medicine, National Defense Medical Center, No.325, Sec. 2, Chenggong Rd., Neihu Dist., Taipei City, 11490, Taiwan, tsghccwang@gmail.com*

***Corresponding author:** Chih-Chia Wang

Department of Family and Community Medicine, Tri-Service General Hospital and School of Medicine, National Defense Medical Center  
No.325, Sec. 2, Chenggong Rd., Neihu Dist., Taipei City, 11490, Taiwan 
Phone No: +886-2-8792-3311 
Fax No: +886-2-8792-7207 
Email Address: [tsghccwang@gmail.com](mailto:tsghccwang@gmail.com)

Contents

Figure S1. Mediation process for medical disputes in the civil legal system in Taiwan ………1

Figure S2. Screening process of the students’ feedback ………………………………………2

Table S1. Evaluation form for court-based learning …………………………...………………3


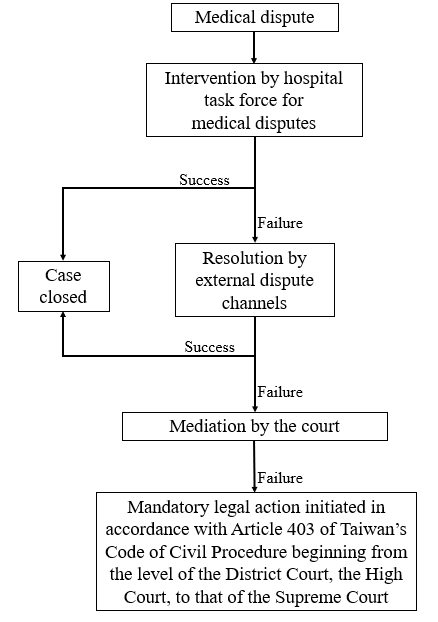


Figure S1. Mediation process for medical disputes in the civil legal system in Taiwan


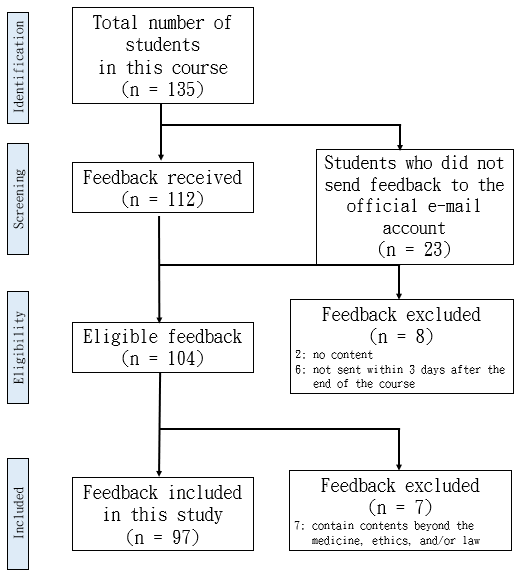


Figure S2. Screening process of the students’ feedback

Table S1. Evaluation form for court-based learning

Part 1a Self-Evaluation of Satisfaction of CBL

|  | Satisfaction | | | | |
| --- | --- | --- | --- | --- | --- |
|  | Highly Dissatisfied | Dissatisfied | Neutral | Satisfied | Highly Satisfied |
| Introduction I |  |  |  |  |  |
| Introduction II (role-play) |  |  |  |  |  |
| Audit a court case |  |  |  |  |  |
| Panel discussion I (Q&A) |  |  |  |  |  |
| Panel discussion II (sharing of experience) |  |  |  |  |  |

Part 1b Self-Evaluation of Practicality of CBL

|  | Practicality | | | | |
| --- | --- | --- | --- | --- | --- |
|  | Not at All Useful | Not Useful | Neutral | Useful | Very Useful |
| Introduction I |  |  |  |  |  |
| Introduction II  (role-play) |  |  |  |  |  |
| Audit a court case |  |  |  |  |  |
| Panel discussion I (Q&A) |  |  |  |  |  |
| Panel discussion II (sharing of experience) |  |  |  |  |  |

Part 1c Self-Evaluation of Knowledge, Attitudes, and Skills

|  | Do Not Understand at All | Do Not Understand | Neutral | Understand | Completely Understand |
| --- | --- | --- | --- | --- | --- |
| Court operations |  |  |  |  |  |
| Medical lawsuit |  |  |  |  |  |
| Mediation |  |  |  |  |  |

|  | Strongly Disagree | Disagree | Neutral | Agree | Strongly Agree |
| --- | --- | --- | --- | --- | --- |
| Less worried about medical disputes |  |  |  |  |  |
| Accept the court’s ruling |  |  |  |  |  |
| Aware of potential medical disputes |  |  |  |  |  |
| Able to show empathy and apply mediation skills |  |  |  |  |  |
| More interested in medical law courses |  |  |  |  |  |

Part 2 Please answer the following:

(1) What did you learn today?

(2) What impressed you most today?
